# Microfluidic Assaying of Circulating Tumor Cells and Its Application in Risk Stratification of Urothelial Bladder Cancer

**DOI:** 10.3389/fonc.2021.701298

**Published:** 2021-06-10

**Authors:** Guanghou Fu, Kok Suen Cheng, Anqi Chen, Zhijie Xu, Xiaoyi Chen, Junjie Tian, Congcong Xu, Yukun Sun, Kuang Hong Neoh, Yun Dai, Ray P. S. Han, Baiye Jin

**Affiliations:** ^1^ Department of Urology, The First Affiliated Hospital, Zhejiang University School of Medicine, Hangzhou, China; ^2^ Jiangzhong Cancer Research Center, Jiangxi University of Traditional Chinese Medicine, Nanchang, China; ^3^ Department of Material Science and Engineering, College of Engineering, Peking University, Beijing, China

**Keywords:** circulating tumor cells, microfluidics, bladder cancer, risk stratification, biomarker

## Abstract

Bladder cancer is characterized by its frequent recurrence and progression. Effective treatment strategies need to be based on an accurate risk stratification, in which muscle invasiveness and tumor grade represent the two most important factors. Traditional imaging techniques provide preliminary information about muscle invasiveness but are lacking in terms of accuracy. Although as the gold standard, pathological biopsy is only available after the surgery and cannot be performed longitudinally for long-term surveillance. In this work, we developed a microfluidic approach that interrogates circulating tumor cells (CTCs) in the peripheral blood of bladder cancer patients to reflect the risk stratification of the disease. In a cohort of 48 bladder cancer patients comprising 33 non-muscle invasive bladder cancer (NMIBC) cases and 15 muscle invasive bladder cancer (MIBC) cases, the CTC count was found to be considerably higher in the MIBC group compared with the NMIBC group (4.67 *vs.* 1.88 CTCs/3 mL, P=0.019), and was significantly higher in high-grade bladder cancer patients verses low-grade bladder cancer patients (3.69 *vs.* 1.18 CTCs/3mL, P=0.024). This microfluidic assay of CTCs is believed to be a promising complementary tool for the risk stratification of bladder cancer.

## Introduction

Bladder cancer is the second most common urogenital malignancy and ranks 13th in the death rate worldwide (1). While around 80% patients were initially diagnosed with non-muscle invasive bladder cancer (NMIBC) (2), over 45% of them experienced tumor recurrence within 2 years and 6% worsen with increased tumor grade. Additionally, 10% of the NMIBC patients may progress to muscle invasive bladder cancer (MIBC) (3), of whom approximately 50% were threatened by remote metastasis even though radical cystectomy has been performed (4, 5). Therefore, patients with bladder cancer require long-term monitoring and surveillance.

Muscle invasiveness and tumor grade are the two critical prognostic factors that clinicians rely on in an attempt to individualize and provide effective treatments. Imaging techniques such as CT and MRI can provide preliminary information of the muscle invasiveness but are impeded by subjective judgement and lack of accuracy (6). Histological biopsy, although regarded as the gold standard for determine both tumor stage and grade, is only available after surgery and is absent in the subsequent long-term follow-up duration. Hence, a timely and easy-to-perform complementary technique is highly necessary to reflect the risk stratification of bladder cancer patients.

Liquid biopsy techniques in recent years has emerged as a promising complementary to traditional diagnostic approaches (7), one example would the wide exploration of circulating tumor cells (CTCs). CTCs in peripheral blood are shed directly from the primary tumor and serve as the delivery vehicles for cancer metastasis, providing the first-hand tumor information about the phenotypic and functional characteristics (8). Interrogating CTCs in cancer patients is on the frontier of next generation diagnosis for the early detection of cancer, the monitoring of disease activity, the evaluation of therapeutic efficacy, as well as the recognition of molecular changes in clonal evolution (9–12). In addition, CTCs enumeration as a prognostic marker have been shown to have significant correlations with disease-free progression and overall survival in various cancers (13–16). Nevertheless, the applicability of CTCs in the clinic is still challenged by their rarity (1–10 CTCs/billions of peripheral blood cells) and heterogeneity.

To date, several technologies for the isolation and enrichment of CTCs have been developed (17). For example, the CellSearch^®^ system (Veridex LLC, Warren, NJ-USA) is approved by the Food and Drug Administration (FDA) for CTC enumeration in metastatic colorectal (18), breast (19) and prostate cancer (20). However, the complexity of the immunomagnetic methodology and the high cost of the reagent kits limit its wide use.

Microfluidic technology has become a low-cost and efficient alternative for the purpose of CTCs isolation (21–24). Some microfluidic approaches achieved a “positive” capture of CTCs based on the antibody coatings at the inner wall of the chip (21, 25, 26) whereas others have proposed a “negative” enrichment of CTCs by eliminating the background cells (27, 28). Despite of the desirable sensitivity, these methods are still not commonly preferred owing to the complicated chip fabrication process.

In an attempt for the in-depth investigation of rare tumor cell in human body fluid, our group previously reported a microfluidic chip that was able to detect urinary exfoliated tumor cells (UETCs) in the urine of the bladder cancer patients (29). In the current work, we further upgraded the microfluidic chip for the label-free isolation of CTCs from the peripheral blood of bladder cancer patients. Taking advantage of the fact that CTCs are usually bigger and less deformable than background blood cells, we captured CTCs with high efficiency and purity. By using the immunofluorescent biomarkers of Pan-CK, CD45 and DAPI, we were able to identify and enumerate CTCs accurately. Furthermore, on the basis of a clinical study involving 48 bladder cancer patients, the correlation between CTCs count and the prognostic factors has been investigated and established. This study showed microfluidic assay of CTCs holds the promise of a robust technique for the risk stratification of bladder cancer patients.

## Materials and Methods

### Fabrication of the Microfluidic Chip

To achieve an efficient isolation of CTCs, a microfluidic chip was specially designed and fabricated. The AutoCAD software (Autodesk Inc.) was used to depict the characteristic design of the microstructures and the microchannels. Following that is a soft lithography process that photoengraved the designed pattern onto a silicon wafer spun with a 20 µm thick layer of SU-8 photoresist. Polydimethylsiloxane (PDMS, Sylgard 184, Dow Corning) constituted the matrix material of the microfluidic chip. Typically, the liquid PDMS was pre-mixed with its curing agent at a ratio of 10:1. The mixture was then degassed for 10 min and casted onto the silicon wafer, followed by the curing process for 4h at 60°C. Afterwards, the PDMS was peeled off from the mold, punched with inlet and outlet holes, treated with oxygen plasma, and bonded to a clean glass slide to form a finished microfluidic chip.

### Evaluation of the Microfluidic Chip Performance

The first is to characterize the capture efficiency of the microfluidic chip, which reflects the ability of isolating cells from the fluid flow. Capture efficiency is defined as the ratio of the number of cells captured by the chip to the number of total input cells. T24 cells were initially stained with CellTracker Red CMTPX Dye (Invitrogen) and diluted several times in a 96-well plate until the concentration reached roughly 200 cells/well. The exact number was counted after the microscopic check and these were regarded as the input cells. The input stained cells were spiked into 3 ml prepared blood sample from healthy donators and then processed by the microfluidic chip. With the enumeration of captured cells, the capture efficiency could be calculated. Different flow rate groups were investigated ranging from 500 ul/h to 3000 ul/h. Five repeated tests were conducted for each group.

The second issue is to assess the inter-assay viability of the microfluidic immunoassay. T24 cells were stained with an immunofluorescent assay (DAPI+/CK20+/CD44v6+) and diluted into 5 groups with different cell concentration, respectively, 5, 10, 20, 50, 100 cells/3ml. For every group, 3 repeats were performed. After processing with the microfluidic chip, the corresponding capture efficiency was calculated. The inter-assay viability are represented by the relative standard deviation (RSD) of the capture efficiency, where

RSD=(Standard deviation)/Mean×100%

### Setup of the Microfluidic-Assay System

The microfluidic-assay system consists of three parts. The first part refers to the microscopic vision module which comprises an inverted microscope (IX, Olympus) and a CCD linked to a Window PC responsible for collecting microscopic images. The second part is the flow control module which includes a syringe pump (SP3D EX, Mindray) and bio-compatible tubing for the transfer of samples. The third part is the fabricated microfluidic chip as aforementioned. To eliminate the possibilities of air bubbles inside the microchannel, the microfluidic chip was pre-flushed with phosphate buffered saline (PBS, Wisent) with 8 mM ethylenediaminetetraacetic acid (EDTA, Wisent) prior to sample processing.

### Ethics and Enrollment of Patients

This research was conducted under the approval of the Ethics Committee of the First Affiliated Hospital at Zhejiang University School of Medicine (Registration No. 2015-218) and complied with the Declaration of Helsinki. 48 bladder cancer patients were enrolled and anonymously indexed from November 2016 to October 2017 with informed consent obtained. All the patients were diagnosed positive by cystoscopy. With the postoperative pathology, 33 patients were confirmed as non-muscle invasive bladder cancer (NMIBC) whereas 15 patients were diagnosed as muscle invasive bladder cancer (MIBC). The mean age of the two groups were 65.7 ± 10.2 and 65.6 ± 10.2 years old, respectively. Histologic assessment was performed by two certified cytopathologists according to the 2004 WHO classification.

### Preparations of Blood Sample

Blood samples of 3 ml per patient were collected in EDTA-coated vacutainer tubes to avoid coagulation. The preoperative and postoperative blood samples were respectively collected on the second morning after hospitalization and after surgery. The samples were sent to the lab within 4h after collection. The vacutainer tube containing the blood was centrifuged at 800g for 5 min and the supernatant serum was discarded. PBS buffer of v/v 1:1 was added to the pellet and pipetted gently. The prepared sample was then subjected to the following microfluidic analyses.

### Microfluidic Assaying of CTCs

The prepared blood sample was driven into the microfluidic chip by the syringe pump at a flow rate of 2 ml/h. Usually, the capture procedure would be finished within 1.5 h. By sequentially introducing solvents into the chip, the captured cells were fixed with 4%-paraformaldehyde (PFA) (Sangon) for 15 min, permeabilized with 0.1% Triton X-100 (Sangon) in PBS for 15 min and incubated with 5% bovine serum albumin (BSA) (Sangon) for 30 mins to reduce non-specific bindings. Pan-CK and CD45 primary antibodies (Abcam) were diluted to a proper concentration according to the manufacturer’s protocol and pumped into the microfluidic chip with a 60 min incubation at room temperature. They were used to mark epithelial cells and white blood cells, respectively. Afterwards, secondary antibodies conjugated with Alexa Fluor 488 and Alexa Fluor 594 (Invitrogen) were introduced into the chip with another 60 min incubation at room temperature, followed by a 20 min incubation with 4’,6-diamidino-2-phenylindole (DAPI) (Invitrogen) to stain the nucleus. The staining process was completed by flushing the chip with PBS to clean the unbonded reagents. In order for a gentle incubation procedure, the flow rate of the all the reagents was set at 500 ul/h. A putative CTC should possess the following features: clear nucleus morphology (DAPI+), epithelial origin (PAN-CK+) and exclusion of the interference from white blood cell (CD45-).

## Results

### Design and Performance Evaluation of Microfluidic Chip

**Figure 1:**
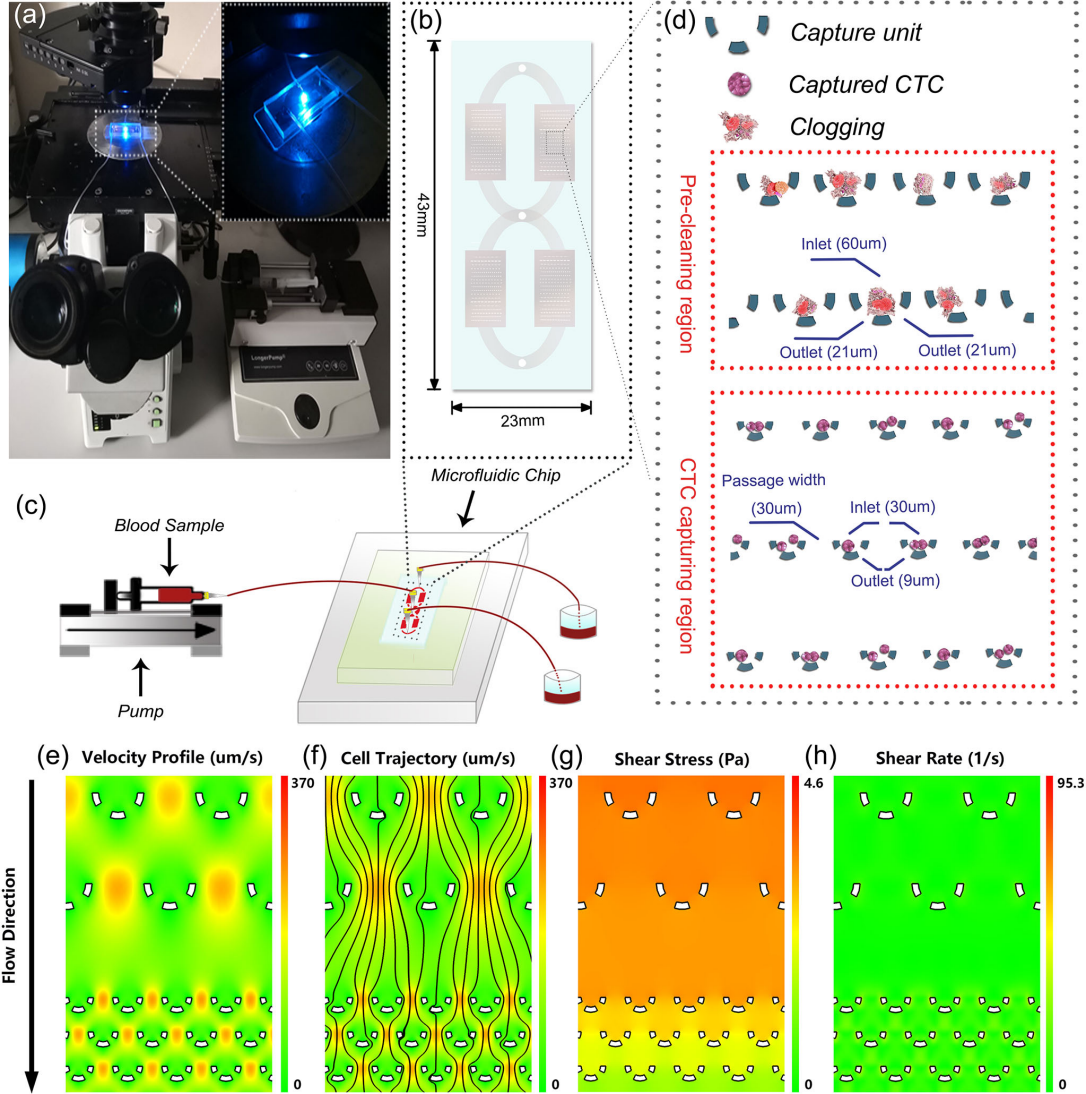
System setup and microfluidic chip design. **(A)** Lab-based setup of the microfluidic system. **(B)** An overview of the microfluidic chip. **(C)** Illustration of the experimental diagram. **(D)** Detailed design of the microfluidic chip. **(E–H)** Numerical simulation of the hydrodynamics in the microfluidic chip.

The microfluidic-assay system was constructed according to **Figures 1A–C**, of which the self-designed microfluidic chip acted as the core component. The basic capture unit of the microfluidic chip is roughly a semicircular arc of three independent micro-pillars (**Figure 1D**). The upward and the bottom openings are respectively the inlet and the outlet of the fluid flow. The outlet size was specially set at 9 um which allows the smaller background cells to pass through while keeping the larger CTCs stuck. To minimize the detrimental clogging commonly encountered in the blood sample, we proposed two solutions. The first was to set dozens of rows of larger capture unit (with an inlet of 60 um and two outlets of 21 um) at the very beginning of the flow pathway. Once the clogging occurs, the debris will be captured and cleaned prior to entering the CTC capturing region. The second was to enlarge the flow passage width between two adjacent capture units to 30 um. Although CTCs might be missed due to wider flow passage, the capture probability can still be compensated and enhanced by having more rows of the capture units. In the optimized design of the microfluidic chip, over 200 rows of the capture units were set in a parallel and staggered manner.

To assess the impact of hydrodynamics on the captured cells, we performed a numerical simulation to reveal the fluidic characteristics. As illustrated int **Figures 1E–H**), the velocity profile and the probable cell trajectory were depicted to reflect a probable capture of the cells. Further analyses showed that in the microchannel, the shear stress caused by the flow ranged from 0 to 4.6 Pa and the shear rate was consistently smaller than 95.3 1/s. These values were within the safe range of human’s normal physiological state of <7.0 Pa stress (30) and <2000 1/s shear rate (31), respectively. These observations convinced us that the microfluidic chip was capable of isolating CTCs in a harmless and intact way.

**Figure 2:**
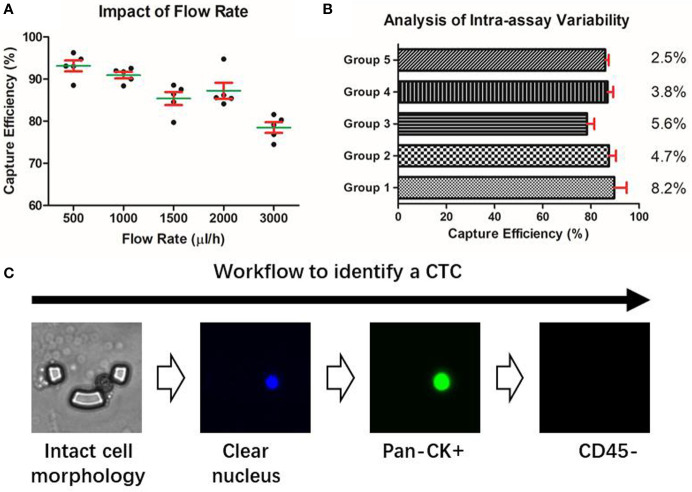
Characterization of the microfluidic approach. **(A)** Capture efficiency of the microfluidic chip. **(B)** Intra-assay variability of the microfluidic assay under different cell concentration groups. **(C)** Identification of a putative CTC based on the microfluidic method.

The performance of the microfluidic chip was characterized from two aspects: the capture efficiency and the inter-assay variability. In spiked cell line experiments, the capture efficiency gradually declined with the increase of the flow rate, but in all the groups, the chip’s capture efficiencies were consistently higher than 75% (**Figure 2A**). To balance the capture efficiency and the time consumption, we chose the flow rate of 2ml/h for the subsequent processing of patients’ blood samples. Further, the experiments conducted on the five groups with different cell concentrations ranging from 5 to 100 cells/3ml showed our method achieved an RSD smaller than 10% (**Figure 2B**), indicating a desirable consistency between assay repeats and a reliable experimental result.

### Identification of CTCs by Immunofluorescent Staining

Due to the epithelial origin of bladder cancer (32), an epithelial marker is capable of distinguishing the CTCs of the bladder tumor from the non-epithelial background blood cells. Pan-CK is a subgroup of intermediate filament proteins, characterized by the diversity and abundance of polypeptides presented in human epithelial tissues (33). Using anti-Pan-CK antibody as a biomarker would be amply adequate to realize a wide coverage recognition of bladder CTCs. Besides, CD45 has been well confirmed as a reliable marker of the white blood cells and DAPI is widely used to stain the nucleus (34). Therefore, a combined marker-panel of “DAPI+/Pan-CK+/CD45-/” enabled us to identify CTCs in the peripheral blood and to eliminate the interference caused by background blood cells (**Figure 2C**).

**Figure 3:**
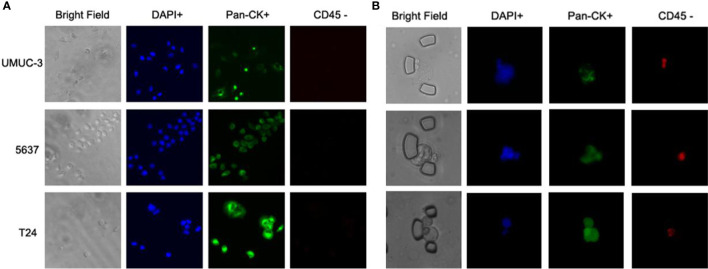
Immunofluorescent test on cell lines and validation on the bladder cancer patients. **(A)** Immunofluorescent staining on three bladder cancer cell lines. **(B)** Captured CTCs from the bladder cancer patients.

To validate the performance of the combined marker-panel, three different human bladder cancer cell lines (UMUC-3, 5637 and T24) were tested. The expected staining and identification of all the cell lines verified the efficacy of our immunofluorescent protocols (**Figure 3A**).

### Correlation Between CTC Enumeration and the Clinical Outcomes of Bladder Cancer

With the successful isolation of CTCs from the peripheral blood (**Figure 3B**), correlations between CTC enumeration and clinical prognostic outcomes were assessed based on a cohort of 48 bladder cancer patients with varied degrees of disease progression. The baseline demographics and clinicopathological characteristics of eligible patients are summarized in **Table 1**.

**Table 1:** Baseline clinicopathological characteristics of the cohort.

Characteristics	NMIBC^1^ (n=33)	MIBC^2^ (n=15)	P-value
**Age, mean (SD)**	65.7 (10.2)	65.6 (10.2)	0.493
**Gender, n (%)**			0.143
Female	2 (6.1)	3 (20.0)	
Male	31 (93.9)	12 (80.0)	
**Body mass index, mean (SD)**	24.1 (3.5)	24.4 (3.7)	0.654
**Smoking history, n (%)**			0.834
Yes	23 (69.7)	10 (66.7)	
No	10 (30.3)	5 (33.3)	
**Drinking history, n (%)**			0.875
Yes	14 (42.4)	6 (40.0)	
No	19 (57.6)	9 (60.0)	
**Hematuria, n (%)**			0.688
Yes	20 (60.6)	10 (66.7)	
No	13 (39.4)	5 (33.3)	
**Urine, median (IQR)**			
Leucocyte (/uL)	20.8 (4.5–52.5)	34.6 (6.5–171.7)	0.317
Bacterium (/uL)	42.2 (13.6–317.8)	100.2 (28.1–513.5)	0.247
**Blood, median (IQR)**			
Serum creatinine (umol/L)	81.0 (73.0–93.5)	85.0 (77.0–92.0)	0.456
Serum urea (mmol/L)	5.8 (5.2–6.8)	5.6 (4.8–7.3)	0.841
Serum uric acid (umol/L)	366.0 (287.0–435.5)	344.0 (281.0–380.0)	0.312
**Pathological grade, n (%)**			0.004
PUNLMP	5 (15.2)	0	
Low grade	11 (33.3)	0	
High grade	17 (51.5)	15 (100.0)	
**Initial BC, n (%)**			0.259
Yes	14 (57.6)	6 (40.0)	
No	19 (42.4)	9 (60.0)	
**Tumor focus, n (%)**			0.724
Nonmultifocality	18 (54.5)	9 (60.0)	
Multifocality	15 (45.5)	6 (40.0)	
**Tumor size, n (%)**			0.040
< 20mm	17 (51.5)	3 (20.0)	
≥ 20mm	16 (48.5)	12 (80.0)	
**Surgical options, n (%)**			0.151
Radical Cystectomy	4 (12.1)	5 (33.3)	
TURBT^3^	29 (87.9)	10 (66.7)	
**CTCs/3 mL, median (IQR)**	1.88 (0.76-3.00)	4.67 (1.41-7.93)	0.019

^1^MIBC, muscle-invasive bladder cancer; ^2^NMIBC, non-muscle-invasive bladder cancer; ^3^TURBT, Transurethral resection of bladder tumor.

**Figure 4:**
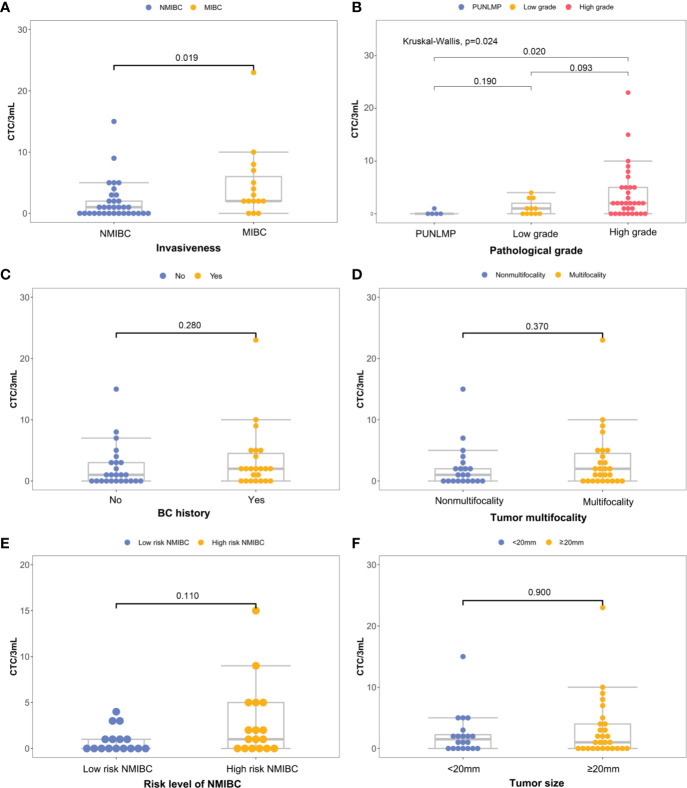
Correlations between CTC count and primary clinical outcomes: **(A)** histological grade, **(B)** invasiveness, **(C)** previous bladder cancer history, **(D)** multifocality, **(E)** progression risks of the NMIBC, **(F)** tumor size.

There is a significant elevation in the CTC count for MIBC versus NMIBC patients [4.67 (95% CI, 1.41-7.93) *vs.* 1.88 (95%CI, 0.76-3.00) CTCs/3 mL; P=0.019] (**Figure 4A**). Similarly, the CTC count increased significantly in the high-grade bladder cancer patients verses the low-grade and PUNLMP (Papillary urothelial neoplasm of low malignant potential) bladder cancer patients [3.69 (95% CI, 1.89-5.49) *vs.* 1.18 (95% CI, 0.19-2.17) *vs.* 0.20 (95% CI, -0.36-0.76) CTCs/3mL; P=0.024]; (**Figure 4B**). By contrast, there were no significant correlations between the CTC enumeration results and other clinical prognostic outcomes such as BC history, tumor multifocality, risk level of NMIBC and tumor size (**Figures 4C–F**).

### CTC Count as a Prognostic Marker of Bladder Cancer

**Figure 5:**
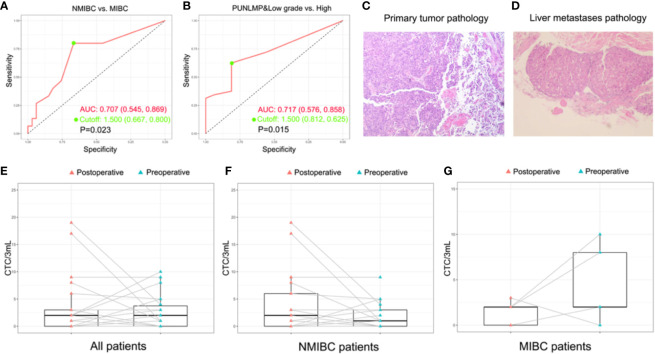
Potential of CTCs as a prognostic biomarker for bladder cancer. **(A, B)** ROC analyses of CTCs as a prognostic biomarker for indicating tumor grade and invasiveness. **(C, D)** The histopathologic result of the primary bladder tumor and the liver metastasis of the patient with remote metastasis. The dynamic change of CTC count after surgery with regard to the whole patient cohort **(E)**, NMIBC group **(F)**, and MIBC group **(G)**.

To assess whether the CTCs count could be used as a supplementary biomarker for the stratification of bladder cancer, we performed ROC analysis of CTC enumeration and patients with bladder cancer in different clinical stages and grades (**Figures 5A, B**). The AUC [95% confidence interval (CI)] were calculated by comparing NMIBC with MIBC group, and high-grade patients with combined PUNLMP and low-grade groups. The AUC in comparing the NMIBC and MIBC cohort was 0.707 (95% CI, 0.545-0.869; P=0.023) with a sensitivity and specificity of 80.0% and 66.7%, respectively (**Figure 5A**). Similarly, the AUC comparing the PUNLMP/low-grade and high-grade cohorts was 0.717 (95% CI, 0.576-0.858; P=0.015) with a sensitivity and specificity of 62.5% and 81.2%, respectively (**Figure 5B**). The optimal cutoffs for distinguishing NMIBC *vs.* MIBC and high-grade *vs.* low-grade bladder cancer were both at 1.5 CTCs/3 mL blood.

## Discussion

Bladder cancer as a global health issue of concern is characterized by the high frequency of recurrence and progression. In current clinical practices, the risk stratification of a bladder cancer patient can only be assessed based on imaging and biopsy results. But the reliance on subjective judgement and the inaccessibility in the long-term follow-up constitute some of the major challenges. Importantly, the exponentially evolving “liquid biopsy” offers the opportunities for low invasive diagnosis, tumor dynamic monitoring and therapy selection. CTCs have been considered a viable and readily accessible alternative source of tumor cells in the form of “liquid biopsy”, which have attracted much attention in bladder cancer research and also have a potential application in clinical diagnosis and prognosis (35, 36). Additionally, unraveling the phenotypic and molecular profile of CTCs provides key information about tumor biology and contributing to individualized precision treatment (37). Nevertheless, the applicability of CTCs as a clinical biomarker has been challenged by their rarity and heterogeneity (38, 39). Numerous approaches (37, 40, 41) for detecting CTCs were proposed but are still not commonly used. This is mainly due to the methodological complexity, the inconsistent readouts caused by the ambiguity of CTC classification and the lack of standard sample preparation (42).

We previously reported a size-based microfluidic chip to efficiently capture and identify urinary-exfoliated tumor cells (UETCs), and predicted objectively the diagnosis and prognosis of bladder cancer patients (29). In this current work, the microfluidic chip was further developed and optimized to specifically detect CTCs. Typically, the size of the basic capture unit in the microfluidic chip was strictly designed in order for an effective distinguishment of CTCs from the background blood cells. To tackle the clogging issue caused by blood coagulation, we divided the microfluidic chip into two functional regions. The first referred to the pre-cleaning region in which enlarged capture units were set to capture clogging and debris while letting cells to pass through. The second region was the CTC capture region where CTCs were isolated based on the size and deformability. To further reduce the clogging effect and improve the processing throughput, we expanded the passage width between the adjacent capture units and compensated the possibility of cell lose by patterning more rows of capture units. Therefore, compared with the conventional microfluidic chips relying on complicated chip fabricating process like antibody coating, our microfluidic chip is developed solely based on the physical properties of the cells and hence, it is a low-cost and user-friendly approach for most of the clinically relevant large-scale studies.

Additionally, the CTCs count was combined with the clinical information for further investigation. With regard to the muscle invasion which had been proved to be an important prognostic factor of bladder cancer (43), CTC count was significantly higher in MIBC patients compared with NMIBC patients (P=0.023). ROC analysis showed that the CTC count as a diagnostic marker achieved a sensitivity of 80.0% and a specificity of 66.7% in differentiating MIBC from NMIBC patients when the cutoff was 1.5 CTC cells/3mL. Similarly, the CTC count was significantly elevated in high-grade bladder cancer patients compared with PUNLMP and low-grade patients (P=0.02) which was also capable of discriminating between the two groups at a diagnostic sensitivity of 62.5% and a specificity of 81.2%. This observation concurs well with the fact that muscle-invasive and high-grade bladder cancer patients are faced with greater risks of metastasis and worse prognosis. Noteworthy, in the total study cohort, one patient (Patient ID No. 12) aroused our great interest during the 3-year follow-up. He was initially presented with gross hematuria and was diagnosed with NMIBC on January, 2017 (**Figure 5C** and **Supplementary Table 1**). At the time of enrollment, his CTCs count was reported at 5 cells/3mL after microfluidic assay, which was apparently higher than that of most other enrolled patients. In the following 3 years, his bladder cancer recurred twice and finally, progressed to liver metastasis on March, 2020 (**Figure 5D** and **Supplementary Figures 1–3**). The above results show that although this patient was initially diagnosed with NMIBC, the comparably higher CTC count coincided well with his subsequent disease progression, indicating that CTC enumeration may serve as a complementary high-risk factor of bladder cancer to guide treatment selection, which has also been verified in other studies (35, 36). In current clinical practices, surgical and therapeutic strategies are largely based on the preoperative prognostic prediction. In other words, a worse prognostic assessment will lead to a more aggressive treatment like radical cystectomy or adjuvant chemotherapy. However, the imaging-based evaluation of muscle invasiveness and the cystoscopy-based biopsy for the preoperative assessment of tumor grade are usually short of accuracy. Therefore, there is an urgent demand for a complementary diagnostic tool that provides clinicians with more accurate information for the disease status. In this sense, CTCs are of no doubt a promising complement.

Furthermore, in a sub-cohort of 22 patients whose paired preoperative and postoperative blood samples were available, we monitored the dynamic change in CTCs count before and after the surgery (**Figure 5E**). Among these patients, 4 out of 5 MIBC patients had a significant decrease in postoperative CTCs count (**Figure 5F**). By contrast, the CTCs count of the remaining 17 NMIBC patients displays a divergent pattern of increase or decrease (**Figure 5G**). Owing to the limited cohort size of our study, we may not draw a solid conclusion on the indication of the postoperative CTCs count, but it is still worthwhile to explore in the future whether the dynamic change of CTCs count reflects the therapeutic and the surgical efficacy of bladder cancer.

Interestingly, in addition to CTCs enumeration, recent advances have been made in unravelling the molecular features of CTCs. Nicolazzo et al. (44) investigated the expression of survivin in the isolated CTCs and found that survivin expression was closed correlated with disease-free survival and cancer-specific survival in NMIBC patients. Similarly, a strong expression of PD-L1 in CTCs was reported to lead to a worse overall survival of patients with urothelial carcinoma (45). What’s more, beyond the scope of CTCs, hemato-chemical biomarkers have also been explored in order for a more accurate risk stratification of bladder cancer. A typical example is basophils, whose absolute count was found closed associated with time to recurrence in high−grade T1 bladder cancer patients (46). Therefore, with the emergence of novel techniques and biomarkers, the management of bladder cancer, especially in the field of NMIBCs, is believed to be developed in a more precise and personalized way.

In summary, we developed a low cost and easy-to-perform microfluidic approach for the isolation and identification of CTCs from bladder cancer patients. The CTCs count was found closely related with several important clinical outcomes including muscle invasiveness and tumor grade, which might facilitate risk stratification evaluation and guide the individualized treatment of bladder cancer in the long-term surveillance. Admittedly, there are still some limitations in our research. On one hand, the throughput and efficiency of the microfluidic approach could be further improved by integrating the lab-based setups into an all-in-one automated system. On the other hand, due to the single-center nature of the study and the limited sample size, our research may not comprehensively reflect the influence of CTCs in bladder cancer. Multi-center clinical trials and inter-laboratory validations involving larger patient cohorts are still needed to verify our findings and promote the clinical translation.

## Data Availability Statement

The raw data supporting the conclusions of this article will be made available by the authors, without undue reservation.

## Ethics Statement

The studies involving human participants were reviewed and approved by the Ethics Committee of the First Affiliated Hospital at Zhejiang University School of Medicine. The patients/participants provided their written informed consent to participate in this study.

## Author Contributions

Conceptualization, BJ and RH. Methodology, GF and AC. Software, K.C. Validation, AC, ZX and XC. Investigation, JT, CX, YS and KN. Resources, YD. Supervision, BJ and RH. Project administration, BJ and RH. Funding acquisition, BJ. All authors contributed to the article and approved the submitted version.

## Funding

This project was supported by the Key Project of the Science and Technology Program of Zhejiang Province, Grant No: 2020C03026 and 2014C03028; and the National Natural Science Foundation of China, Grant No: 81902604.

## Conflict of Interest

The authors declare that the research was conducted in the absence of any commercial or financial relationships that could be construed as a potential conflict of interest.
