# Supplementary material for: Microfluidic Assaying of Circulating Tumor Cells and Its Application in Risk Stratification of Urothelial Bladder Cancer
Source: Front Oncol. 2021 Jun 10;11:701298. doi: 10.3389/fonc.2021.701298 (PMC8222714; doi:10.3389/fonc.2021.701298)
Supplement: Supplementary file 1 [file Table_1.docx]

**Supplementary Materials**

**Supplementary Materials and Methods**

Numerical simulation of the flow pattern in the microfluidic chip

The hydrodynamic profile of the chip was simulated using COMSOL Multiphysics software (COMSOL Inc.). The flow velocity, shear stress and the shear rate inside the microfluidic chip were specially considered. A laminar flow model was adopted to mimic the flow pattern. Human blood, with the density of 1.05 g/cm3 and viscosity of 7.3 mPa·s, was analyzed as the matrix fluid at a flow rate of 2 ml/h.

Cell line culture

Bladder cancer cell lines with varied degrees of differentiation were cultured, respectively, T24, 5637, and UMUC-3 cells. Cells were obtained from Type Culture Collection of the Chinese Academy of Sciences, China, and have passed the STR DNA profiling and mycoplasma contamination check. All the cells were cultured in RPMI-1640 Medium (Gibco) supplemented with 10% FBS (Gibco) at an atmosphere of 37 ℃ comprising 5% CO2. Prior to the experiments, confluent cells were disassociated with trypsin (Gibco), washed twice with PBS, followed by being spiked PBS for subsequent use. In order for a reliable and consistent study, cells within 10 passages after thawing were preferred.

Statistical analysis

The R version 4.0.2 software (Institute for Statistics and Mathematics, Vienna, Austria; https://www.r-project.org/) was used to perform data statistical analyses. The Kolmogorov-Smirnov test was applied to detect the normal distribution of all data. When the data was normally distributed, it was presented as the mean (SD), otherwise it was presented as the median (IQR, inter-quartile range). The Fisher's exact test was performed to contrast the class variable among the cohorts. For non-normally distributed CTCs count, the Mann-Whitney test was used to perform the non-parametric test between the two cohorts. The receiver operating characteristic (ROC) curves were evaluated from a bladder cancer cohort that consisted of 33 patients with NMIBC and 15 MIBC. The area under the curve (AUC) was calculated and the optimal cutoff (diagnostic threshold) was determined when the sum of sensitivity and specificity was the maximum. For the correlation between CTCs enumeration and clinical prognostic outcomes, the Wilcoxon signed rank test was used to conduct difference comparisons of two groups whereas the Kruskal-Wallis tests was used to compare three or more groups. The ggplot2 R package (“ggroc” and “ggplot” function) was employed to calculate and visualize the correlation between CTCs enumeration and risk stratification in bladder cancer cohort. Furthermore, the ggplot2 R package was also used to compare the result of CTCs enumeration between preoperative and postoperative for several bladder cancer patients. Statistical significance was considered when a two-sided P < 0.05 was obtained.

**The background information of Patient No.12 (a metastatic case)**

**Supplementary table 1. The clinical information of Patient No.12 (a** **metastatic case)**

| **Characteristics** | **clinical results** |
| --- | --- |
| Gender | Male |
| Age (years) | 65 |
| Height (cm) | 165 |
| Weight (kg) | 75 |
| BMI (kg/m2) | 27.5 |
| Urine leucocyte (/uL) | 146.4 |
| Urine bacterium (/uL) | 168.9 |
| Serum creatinine (umol/L) | 83 |
| Serum urea (mmol/L) | 5.7 |
| Serum uric acid (umol/L) | 242 |
| Smoking history | None |
| Drinking history | None |
| Initial BC | Yes |
| Hematuria | Yes |
| Tumor focus | Multifocality |
| pT (8th TNM staging) | T1 |
| Grade (WHO 2004) | High grade |
| Preoperative CTCs (/3 mL) | 5 |
| Postoperative CTCs (/3 mL) | 0 |
| BC, bladder cancer; BMI, body mass index; TNM, tumor node metastasis; WHO, world health organization; CTCs, circulating tumor cells | |


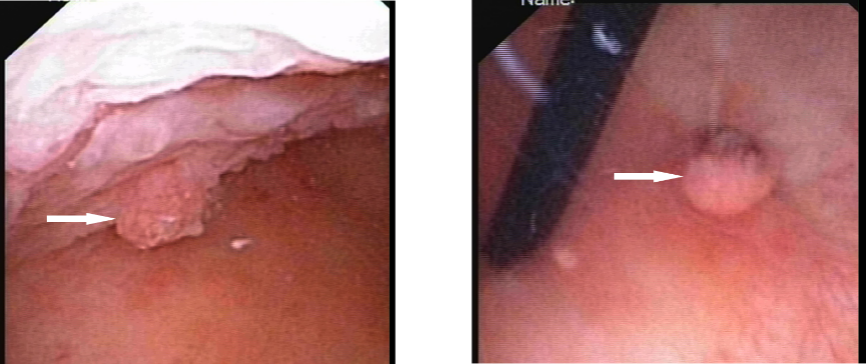


Supplementary Fig. 1 The cystoscopic imaging of the patient at initial diagnosis


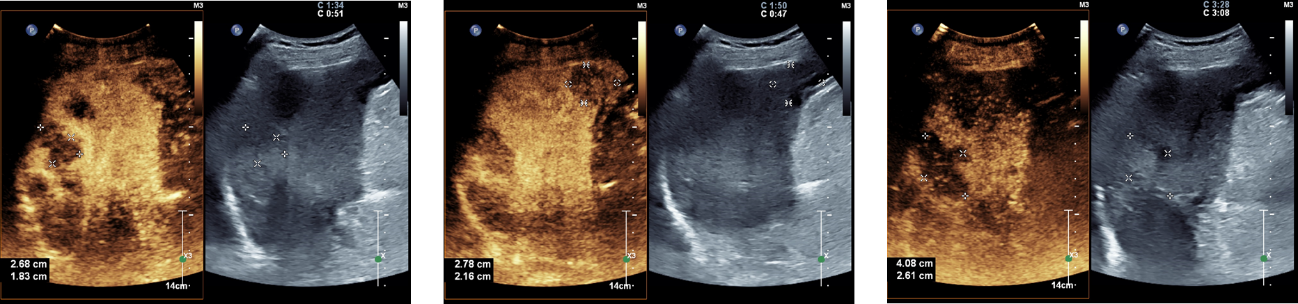


Supplementary Fig. 2 The ultrasound imaging of metastatic foci in the liver


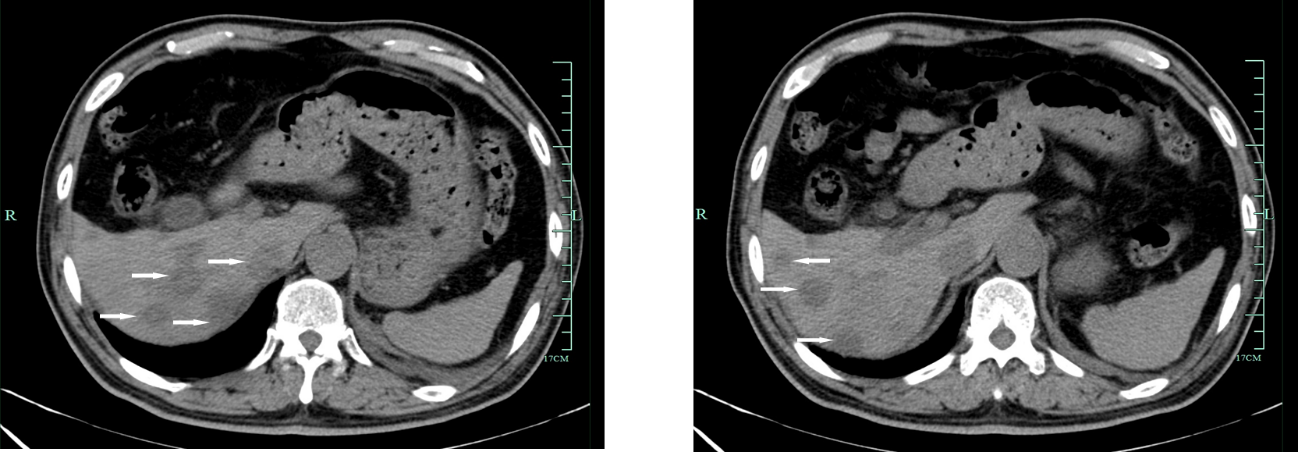


Supplementary Fig. 3 The CT imaging of metastatic foci in the liver
